# Femoral placement of a totally implantable venous access port with spontaneous catheter fracture: case report

**DOI:** 10.1186/s42155-019-0094-9

**Published:** 2020-01-06

**Authors:** Tomohiro Kondo, Shigemi Matsumoto, Keitaro Doi, Motoo Nomura, Manabu Muto

**Affiliations:** Department of Therapeutic Oncology, Graduate School of Medicine, Kyoto University, 54 Shogoin-Kawaharacho, Sakyo-ku, Kyoto, 606-8507 Japan

**Keywords:** Totally implanted venous access port, Late complication, Catheter fracture, Femoral vein

## Abstract

**Background:**

The incidence of catheter fracture after standard positioning of a totally implantable venous access port (TIVAP) is reported to be 1.1%–5.0%; however, the incidence of catheter fracture after TIVAP implantation at a femoral site remains unclear.

**Case presentation:**

In a 30-year-old man with angiosarcoma of the right atrium, tumor embolism was observed from the left brachiocephalic vein to the superior vena cava. A TIVAP was implanted in the right femur. A catheter fracture was spontaneously observed after 7 months.

**Conclusions:**

To the best of our knowledge, this is the first case of catheter fracture in a TIVAP implantation at a femoral site.

## Background

The totally implantable venous access port (TIVAP) is widely used for drug access or infusion during cancer treatment (Kurul et al. 2002). As a standard implantation position, TIVAP is usually inserted via the left or right internal jugular vein (IJV) or subclavian vein and implanted in the forearm, upper arm, or chest (Goltz et al. 2014; Kurul et al. 2002). In patients with central vein occlusions, bilateral breast cancer, infection, cutaneous metastasis, or radiogenic dermatitis, femoral placement of TIVAP is considered as an alternative implantation position (Almasi-Sperling et al. 2016; Bertoglio et al. 1996; Chen et al. 2008; Goltz et al. 2014; Kato et al. 2016; Wolosker et al. 2004).

Patients with TIVAP are at risk for late complications. Late complications of TIVAP mainly include catheter-related infection, catheter occlusion, catheter-related thrombosis, and catheter fracture. Several recent studies reported that the incidence of catheter fracture in a standard position was 1.1%–5.0% (Kurul et al. 2002). However, the incidence of catheter fracture in the patients with femoral placement of TIVAP has not been reported (Almasi-Sperling et al. 2016; Bertoglio et al. 1996; Chen et al. 2008; Goltz et al. 2014; Kato et al. 2016; Wolosker et al. 2004). To the best of our knowledge, here, we report the first case of angiosarcoma in a patient who developed a complication of spontaneous catheter fracture following TIVAP implantation in the femoral vein.

## Case presentation

**Fig. 1:**
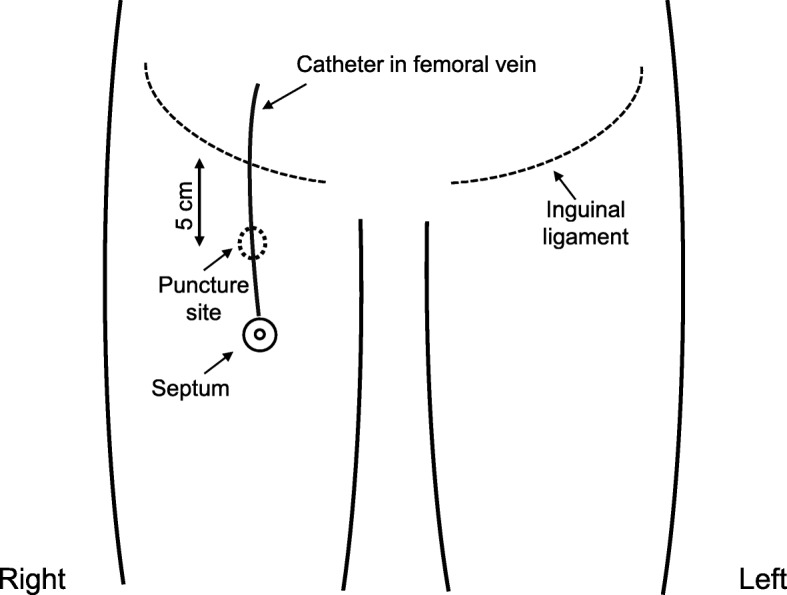
The implantation site of totally implantable venous access port (TIVAP). TIVAP was implanted via the right femoral vein by ultrasound-guided procedure with “Out-of-plane” puncture. The right femoral vein was punctured percutaneously about 5 cm distal to the inguinal ligament. The TIVAP septum was placed in a subcutaneous pocket at the proximal anterior thigh

**Fig. 2:**
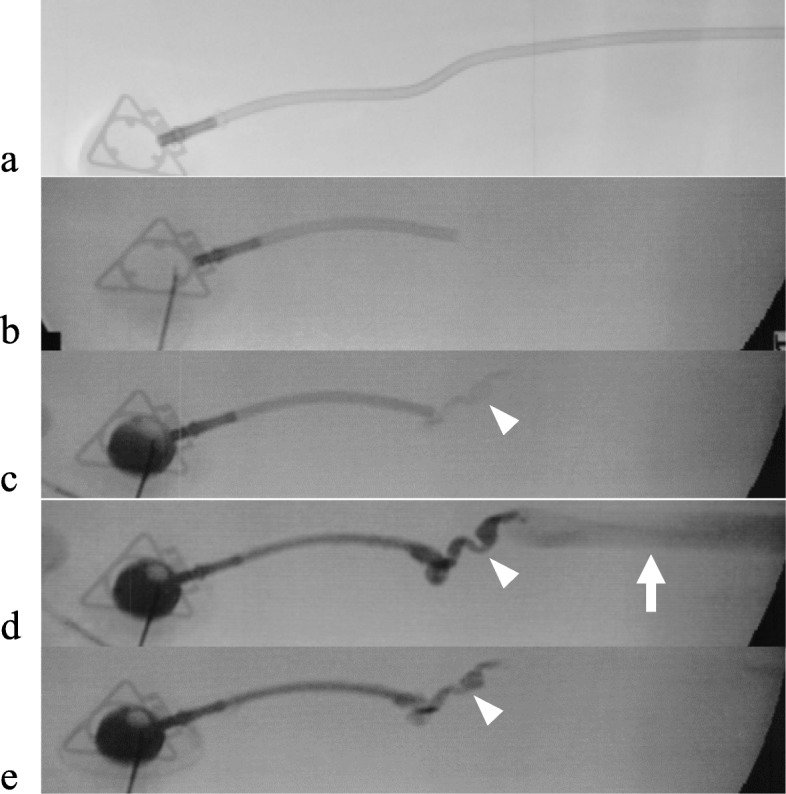
Contrast injection via totally implantable venous access port (TIVAP) with fractured catheter. **a** X-ray photography obtained after implantation of TIVAP. **b** The fractured catheter was spontaneously found 7 months after implantation of TIVAP. **c**, **d**, **e** Saline was injected upon removal of the TIVAP septum; back flow from the TIVAP septum was slightly bloody. When contrast-enhanced sodium was injected through the TIVAP septum, contrast flow was observed in the femoral vein (arrow). A narrow tract can be seen between the tip of the fractured catheter and the femoral vein (arrowhead)

**Fig. 3:**
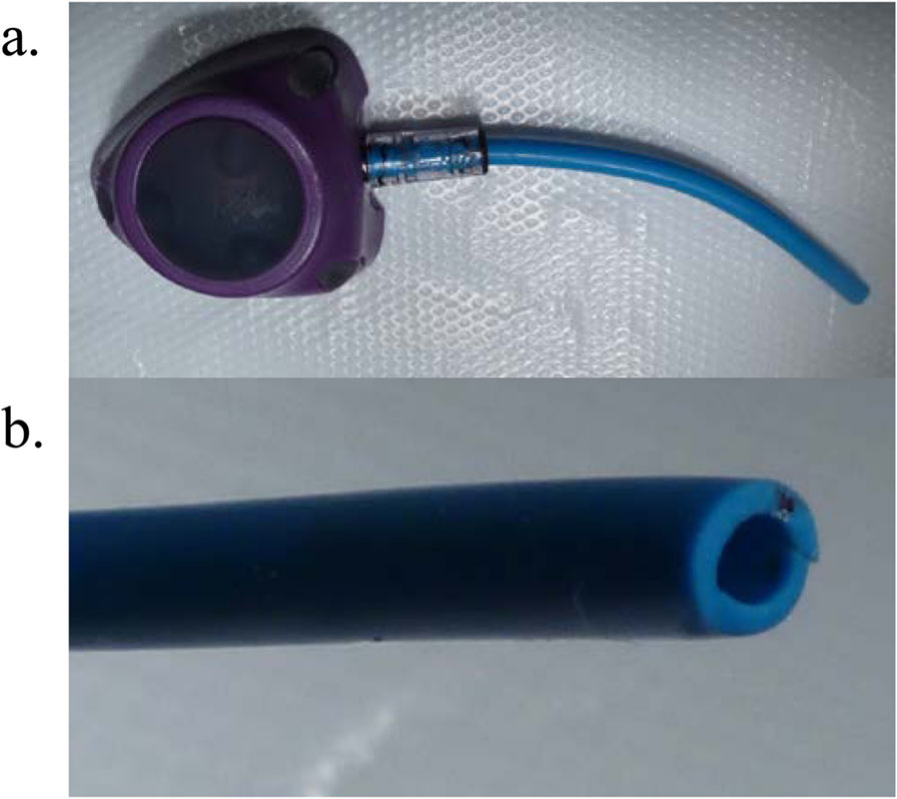
Edges of the fractured catheter. **a** The vertical catheter fracture at a point 4 cm from the connector, which was circumferentially aligned. **b** The edges of the fractured catheter were rounded and polished. Upon removal of the TIVAP septum, the cross-section of the fracture was dull, with an overall elliptical shape

A 30-year-old man visited to our hospital with symptom of chest discomfort. Magnetic resonance imaging revealed a tumor in the right atrium. Biopsy specimens from the tumor revealed angiosarcoma. The tumor was surgically excised and the right atrium was subsequently reconstructed. After surgery, chemoradiotherapy with paclitaxel/carboplatin and radiotherapy (50.4Gy/28Fr) were indicated as adjuvant treatment. Computed tomography (CT) performed 3 years after completion of chemoradiotherapy revealed recurrence of angiosarcoma with enlarged mediastinal lymph nodes, liver metastasis, and tumor embolism. Because the tumor embolism was observed to have traveled from the left brachiocephalic vein to the superior vena cava, a power-injectable Groshong silicone TIVAP (Bard Power Port, Bard Access system Inc., Salt Lake City, UT) was implanted via the right femoral vein by an ultrasound-guided procedure with “Out-of-plane” puncture. The right femoral vein was punctured percutaneously about 5 cm distal to the inguinal ligament. The TIVAP septum was placed in a subcutaneous pocket at the proximal anterior thigh (Fig. 1). The patient’s palliative chemotherapy regimen comprised weekly paclitaxel (80 mg/m^2^ intravenously on days 1, 8, and 15, every 28 days). Seven months after initiating this regimen, CT revealed a catheter fracture. The fractured catheter migrated through the hepatic vein to the left pulmonary artery and it was retrieved by an interventional radiologist without any symptoms of catheter fracture. Upon removal of the TIVAP septum, saline was injected; back flow from the TIVAP septum was slightly bloody. When contrast-enhanced sodium was injected at the TIVAP septum, contrast could be seen flowing in the femoral vein (Fig. 2). The catheter fracture had occurred vertically at 4 cm from the connector, which was circumferentially aligned. The fractured catheter’s edges were found to be rounded and polished and the fracture’s cross-section was found to be dull with an elliptical shape on TIVAP septum removal (Fig. 3).

## Discussion

To the best of our knowledge, this is the first case of catheter fracture occurring during femoral placement of TIVAP. Several retrospective studies reported that femoral placement of a TIVAP was feasible in view of the high technical success rate as well as low early and late complication rates (Almasi-Sperling et al. 2016; Bertoglio et al. 1996; Chen et al. 2008; Goltz et al. 2014; Kato et al. 2016; Wolosker et al. 2004). Even in cases of late complications, infection, catheter occlusion, and blood clots were reported, but not catheter fracture (Almasi-Sperling et al. 2016; Bertoglio et al. 1996; Chen et al. 2008; Goltz et al. 2014; Kato et al. 2016; Wolosker et al. 2004). However, as the catheter fracture in the femoral position has not been previously reported, the reasons why catheter fracture occurred in the femoral placement are unclear.

Then, we consider three plausible reasons contributed to catheter fracture in the femoral vein. The first possibility was that chronic stress caused by motion of the hip joint and thigh. The most common cause of catheter fracture in the standard position is “pinch-off syndrome”. This syndrome occurs when the catheter is compressed between the first rib and the clavicle (Kurul et al. 2002). In the present case, the fractured catheter’s edges were rounded and polished due to repeated material wear, with part of the circumference of the break manifesting a rough/granular texture. These findings indicated that this flexural fatigue damage might result in a complete break. Furthermore, the overall elliptical shape of the fracture cross-section indicated repeated kinking of the tubing.

The second possibility was that silicone catheters are more prone to fracture. A retrospective analysis of 698 consecutively implanted TIVAP at the forearm indicated that fracture of the catheter was observed in 3/302 (1.0%) cases in which a silicone catheter was used, whereas no rupture occurred when a polyurethane catheter was used (Wildgruber et al. 2016). Moreover, in patients with implanted TIVAP at the IJV, Groshong silicone catheters were reported as a potential risk for catheter fracture (Saijo et al., n.d.). Power-injectable Groshong silicone TIVAP could be attributed to the fracture in our case.

The final possibility was ultrasound-guided “out-of-plane” puncture of the femoral vein. We performed “out-of-plane” puncture of the femoral vein, which is reported to be a risk factor for IJV puncture. A retrospective analysis of 338 removed ports reported that out-of-plane” ultrasound-guided puncture of the IJV was significantly associated with catheter ruptures, which is invariably associated with a more vertical pathway and a narrower angle at the entry point into the vein wall (Balsorano et al. 2014).

When the backflow of blood from TIVAP is insufficient, X-ray photography should be considered for early detection of catheter fracture. In the present case, catheter fracture occurred even though injection through TIVAP proceeded smoothly. Contrast-enhanced sodium was injected through TIVAP and sodium flow could be observed in the femoral vein. These atypical findings could be due to a narrow tract between the tip of the fractured catheter and the femoral vein shown by contrast injection (Fig. 2).

In conclusion, we experienced the first case of spontaneous catheter fracture after femoral placement of a TIVAP. This fracture could be explained by chronic stress to the catheter caused by motion and use of silicone catheters and ultrasound-guided “out-of-plane” punctures. Even in cases in which it is possible to injection through TIVAP, catheter fracture should be considered as a possible complication of femoral placement of a TIVAP when the backflow of blood from TIVAP is insufficient. Further investigation is needed to clarify the safety of femoral placement of a TIVAP.

## Data Availability

Not applicable.

## Abbreviations

CT: Computed tomography
IJV: internal jugular vein
TIVAP: totally implantable venous access port
